# Apocynin Treatment Prevents Cardiac Connexin 43 Hemichannels Hyperactivity by Reducing Nitroso-Redox Stress in Mdx Mice

**DOI:** 10.3390/ijms21155415

**Published:** 2020-07-30

**Authors:** Alejandra Z. Vielma, Mauricio P. Boric, Daniel R. Gonzalez

**Affiliations:** 1 Departamento de Fisiología, Facultad de Ciencias Biológicas, Pontificia Universidad Católica de Chile, Santiago 8320000, Chile; alvielma@uc.cl (A.Z.V.); mboric@bio.puc.cl (M.P.B.); 2 Departamento de Ciencias Básicas Biomédicas, Facultad de Ciencias de la Salud, Universidad de Talca, Talca 3460000, Chile

**Keywords:** S-nitrosylation, NADPH oxidase, Duchenne, NOX2, heart

## Abstract

Duchenne muscular dystrophy (DMD) is a fatal disease that causes cardiomyopathy and is associated with oxidative stress. In the heart, oxidative stress interferes with the location of connexin 43 (Cx43) to the intercalated discs causing its lateralization to the plasma membrane where Cx43 forms hemichannels. We tested the hypothesis that in DMD cardiomyopathy, increased oxidative stress is associated with the formation and activation of Cx43 hemichannels. For this, we used mdx mice as a DMD model and evaluated cardiac function, nitroso-redox changes and Cx43 hemichannels permeability. Mdx hearts presented increased NADPH oxidase-derived oxidative stress and increased Cx43 S-nitrosylation compared to controls. These redox changes were associated with increased Cx43 lateralization, decreased cardiac contractility and increased arrhythmic events. Pharmacological inhibition of NADPH oxidase using apocynin (one month) reduced systemic oxidative stress and reversed the aforementioned changes towards normal, except Cx43 lateralization. Opening of Cx43 hemichannels was blocked by apocynin treatment and by acute hemichannel blockade with carbenoxolone. NADPH oxidase inhibition also prevented the occurrence of apoptosis in mdx hearts and reversed the ventricular remodeling. These results show that NADPH oxidase activity in DMD is associated with S-nitrosylation and opening of Cx43 hemichannels. These changes lead to apoptosis and cardiac dysfunction and were prevented by NADPH oxidase inhibition.

## 1. Introduction

Duchenne muscular dystrophy (DMD) is a genetic disease caused by mutations of a gene located in the X chromosome that codifies for dystrophin, a protein that connects the cytoskeleton with the sarcolemma, stabilizing the cell membrane [1]. The lack of dystrophin results in progressive skeletal muscle damage and degeneration [2]. Besides the skeletal muscle complications, patients with DMD and related forms of muscular dystrophy such as Becker dystrophy develop cardiac alterations such as arrhythmias and heart failure. Approximately 20% of DMD patients die around the age of 20, mainly due to severe cardiac complications [3]. Despite important efforts to find an effective treatment for the disease, current therapeutic approaches are mostly palliative: physical therapy, psychomotor support and symptom therapy [4,5]. Dystrophic cardiomyopathy can be treated pharmacologically with angiotensin-converting enzyme (ACE) inhibitors, β-adrenergic blockade and glucocorticoid therapy [6], approaches that ameliorate the symptoms and delay the progression to cardiac failure; however, they neither arrest nor reverse the disease [7].

One of the key features of DMD and other cardiomyopathies is the presence of oxidative stress, i.e., an increase in the production of reactive oxygen species (ROS) [8,9,10]. We and others have shown an important role for the NADPH oxidase system as the probable main source of cardiac ROS in DMD [11,12]. In the heart, two isoforms of NADPH oxidase (NOX) are expressed: NOX2, located in the sarcolemma [13], and NOX4, mainly associated to mitochondria [14].

Oxidative stress is known to alter the function of key proteins in the heart. For instance, in DMD, NOX2 derived-ROS modifies the function of the ryanodine receptor, altering calcium handling in cardiomyocytes [12]. Oxidative stress also alters the subcellular location of connexin 43 (Cx43) in the heart [15,16]. Cx43 is the main component of gap junctions (GJ) that mediate the electrical coupling between adjacent cardiomyocytes. Each GJ is formed by the juxtaposition of two hexameric arrangement of connexins, termed hemichannels (or connexons), one from each cell [17]. In the heart, GJ are located mainly in the intercalated discs, forming low-resistance channels that allow the transmission of the action potential between adjacent cardiomyocytes, allowing the propagation of an excitatory wave, leading to the coordinated contraction of the myocardium syncytium [17]. The presence of oxidative stress disrupts the normal transport of Cx43 to the intercalated disc towards the lateral membrane [15]. This lateralization may compromise the normal flow of the action potentials, creating the substrate for the development of arrhythmias.

In addition to forming GJ, connexins can form hemichannels—unopposed connexons in the plasma membrane [17]. Connexin hemichannels (CH) constitute a permeability pathway for ions and small molecules between the cytoplasm and the extracellular milieu. Cx43 hemichannels are permeable to organic ions and small molecules of up to 1000 Da or 1.5 nm in diameter, such as NAD+ and ATP [18]. Under normal physiological conditions, connexons mostly form GJ, while hemichannels are closed. However, in pathological scenarios or during inflammatory processes, dysregulation of CH may lead to their increased activity, thus promoting the dissipation of ionic gradients across the plasma membrane, ultimately causing cell death [19].

Regarding mechanisms of regulation, it has been shown that Cx43 hemichannels activity is regulated by redox potential, pH and posttranslational modifications such as phosphorylation, N-acetylation and S-nitrosylation [20]. Thus, oxidative stress affects Cx43 hemichannels conductivity and permeability. In the heart, CHs opens during metabolic inhibition or reperfusion after an ischemic event [19]. Accordingly, pharmacological blockade of Cx43 hemichannels has been shown to attenuate damage due to the reperfusion [21]. Until very recently, information regarding the existence and role of Cx43 hemichannels in the dystrophic heart was missing. We reasoned that NADPH oxidase-derived oxidative stress causes deregulation of connexin 43 hemichannels in DMD hearts, contributing to the progression of the disease. Accordingly, we aimed to demonstrate the effect of NADPH oxidase inhibition in Cx43 distribution, S-nitrosylation status and cardiac function in mdx mice, a model of DMD. Our results highlight the relevance of Cx43 S-nitrosylation in mdx cardiac dysfunction and remodeling, and the importance of NADPH oxidase-derived ROS in this process.

## 2. Results

### 2.1. Cx43 is Lateralized and S-Nitrosylated in Mdx Hearts

There is evidence that indicates a relationship between oxidative stress and Cx43 lateralization [15]. Therefore, we decided to investigate the state of NADPH oxidase-derived oxidative stress and its impact on Cx43 subcellular location and redox state in mdx mice, an established animal model for DMD.

**Figure 1:**
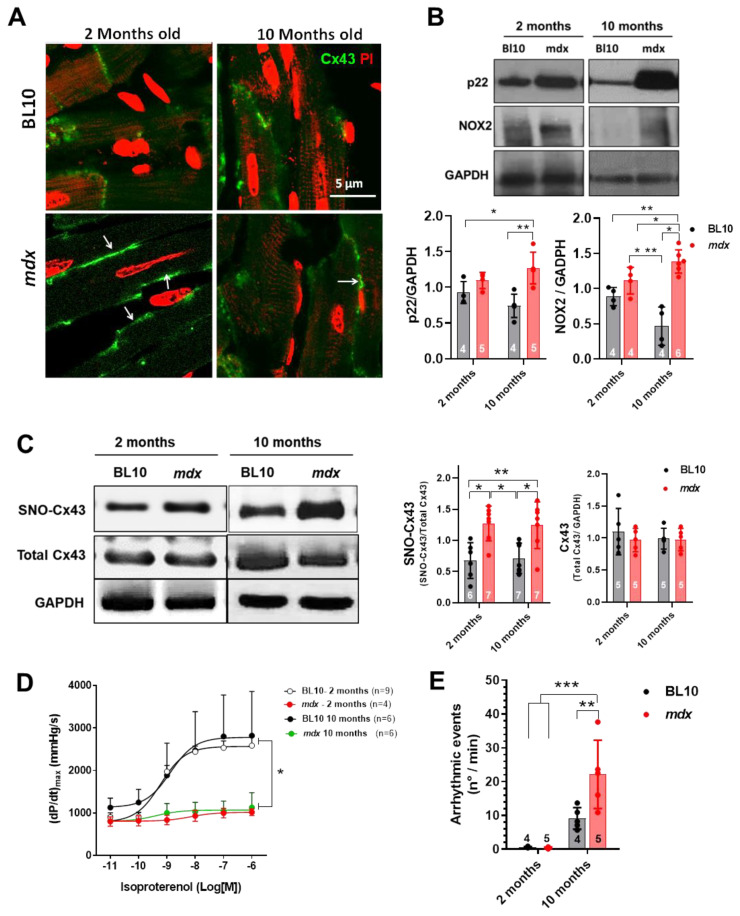
Connexin 43 location S-nitrosylation and NOX2 expression in dystrophic hearts at 2 and 10 months of age. (**A**) Panel of representative confocal images of cardiac sections of control and mdx mice for Cx43 and counterstained for nuclei (propidium iodide, PI). The arrows indicate lateralized Cx43. (**B**) Western blot analysis for the NADPH oxidase-2 subunits p22 and NOX2. The upper panels show representative Western blots for p22, NOX2 and GADPH as loading control. The graphs depict the quantification of p22 and NOX2, normalized to GADPH levels. (**C**) S-nitrosylation of Cx43 in cardiac homogenates assessed by the biotin-switch method. The left panel shows representative Western blots for S-nitrosylated and total Cx43 in 2- and 10-month-old BL10 and mdx hearts. The graphs at the right depict the quantification of nitrosylated and total Cx43. (**D**) Assessment of cardiac contractility evaluated as dP/dt_max_ in isolated hearts from controls (BL10) and mdx mice, two and ten months of age, using increasing concentrations of isoproterenol as adrenergic agonist. (**E**) Evaluation of the occurrence of arrhythmic episodes in the same hearts submitted to isoproterenol perfusion. The *n* is indicated in the bars or the legend of each graph. * *p* < 0.05; ** *p* < 0.005; *** *p* < 0.001 for comparisons indicated by the brackets, ANOVA with Newman–Keuls post-hoc test.

Mdx mice hearts presented increased Cx43 lateralization at 2 and 10 months of age (Figure 1A). Furthermore, as previously described, mdx hearts presented increased expression of the NADPH oxidase subunits NOX2 and p22 compared to age-matched controls (Figure 1B) at both ages. Interestingly, at these ages, mdx hearts also presented increased S-nitrosylation of Cx43 (Figure 1C). Next, we analyzed cardiac function in isolated hearts from control and mdx mice of both ages: contractility and the occurrence of arrhythmias. For this, we stimulated isolated hearts with increasing concentrations of isoproterenol, a β-adrenergic agonist, and evaluated cardiac contractility using dp/dt_max_ as an indicator of inotropic state. While hearts from control genetic background (BL10) mice showed a robust concentration-dependent increase in contractility, dystrophic hearts were unable to respond to the isoproterenol challenge (Figure 1D). Furthermore, the occurrence of arrhythmic episodes during this stimulation protocol was greatly increased in hearts of 10-month-old mdx mice, but not at two months (Figure 1E). Given this clear cardiac phenotype at 10 months of age, we decided to investigate whether NADPH oxidase inhibition could reverse this phenotype by restoring Cx43 location and redox status.

### 2.2. Reduction of Oxidative Stress in Mdx Mice after NOX Inhibition Treatment

We decided to investigate the effect of NOX inhibition in the dysregulation of Cx43 location, activity and redox modification (S-nitrosylation) in dystrophic cardiomyopathy, since there is clear evidence that NOX2 is responsible for the oxidative stress reported in the heart and skeletal muscle of these animals [12,13,22]. For this aim, mice were treated for one month with the vehicle or apocynin, a NADPH oxidase inhibitor. One month treatment with the NOX inhibitor apocynin (40 mg/Kg/day) caused no change in behavior (food and water consumption mainly), nor casualties in any of the groups, confirming the safety of the treatment.

**Figure 2:**
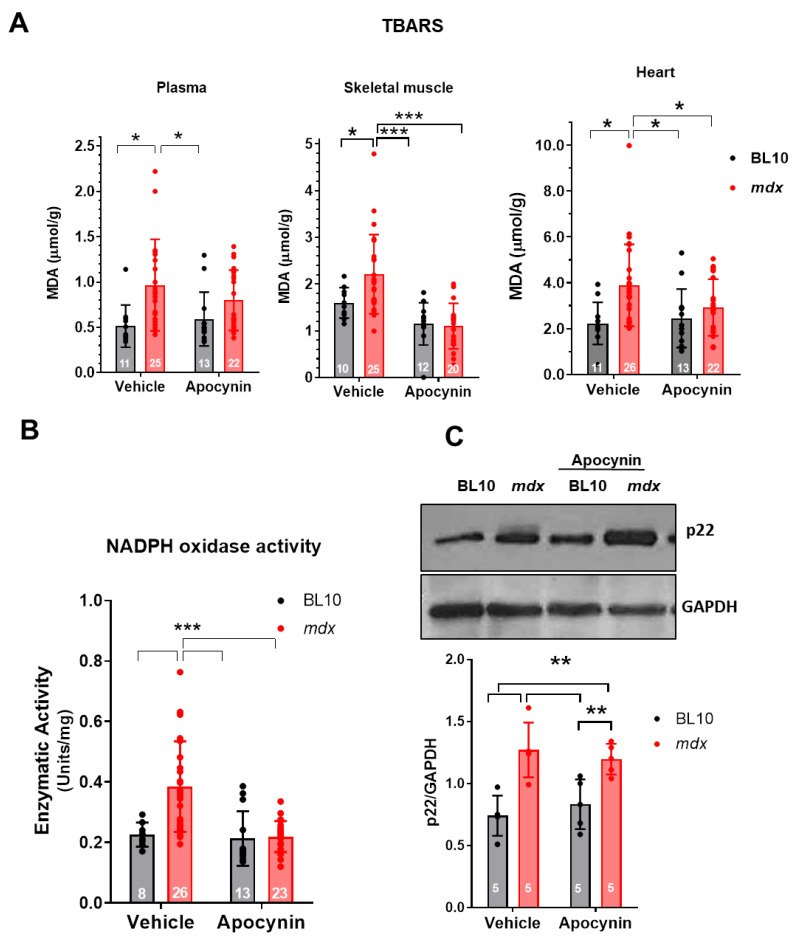
Apocynin treatment reduces NADPH oxidase activity and oxidative stress in dystrophic mice. (**A**) Graphs showing the levels of thiobarbituric acid-reactive substances (TBARS), measured as malondialdehyde (MDA) content in plasma, skeletal muscle and heart from Bl10 and mdx mice, treated with the vehicle or apocynin for one month. (**B**) NADPH oxidase activity, measured as cytochrome-C reduction activity, in cardiac homogenates from BL10 and mdx mice, treated with the vehicle or apocynin. (**C**) Western blot analysis of the levels of p22 in cardiac homogenates from BL10 and mdx mice, treated with the vehicle or apocynin for one month. The *n* is indicated in each bar of the graphs. Asterisks *, **, ***, indicate *p* < 0.05, 0.005 or 0.001 for comparisons the indicated by the brackets, ANOVA followed by Newman–Keuls as post-hoc test.

Next, we analyzed the impact of apocynin treatment on oxidative stress in control and in mdx mice. As a surrogate of oxidative stress marker, we measured the levels of thiobarbituric acid-reactive substances (TBARS) in plasma, skeletal muscle and heart samples. TBARS levels were increased in mdx mice and restored to control values after one month of apocynin treatment (Figure 2A). NADPH oxidase activity determined in heart homogenates was also increased in non-treated mdx mice, but similar to controls (BL10 mice) in apocynin-treated mdx hearts (Figure 2B). As expected, treatment with the inhibitor did not modify the cardiac levels of NADPH oxidase subunit p22, which remain elevated in mdx mice (Figure 2C). Together, these results indicate that one month treatment with apocynin was effective in reducing the oxidative stress present in mdx mice.

### 2.3. Cx43 Hemichannels Activity in Mdx Hearts

**Figure 3:**
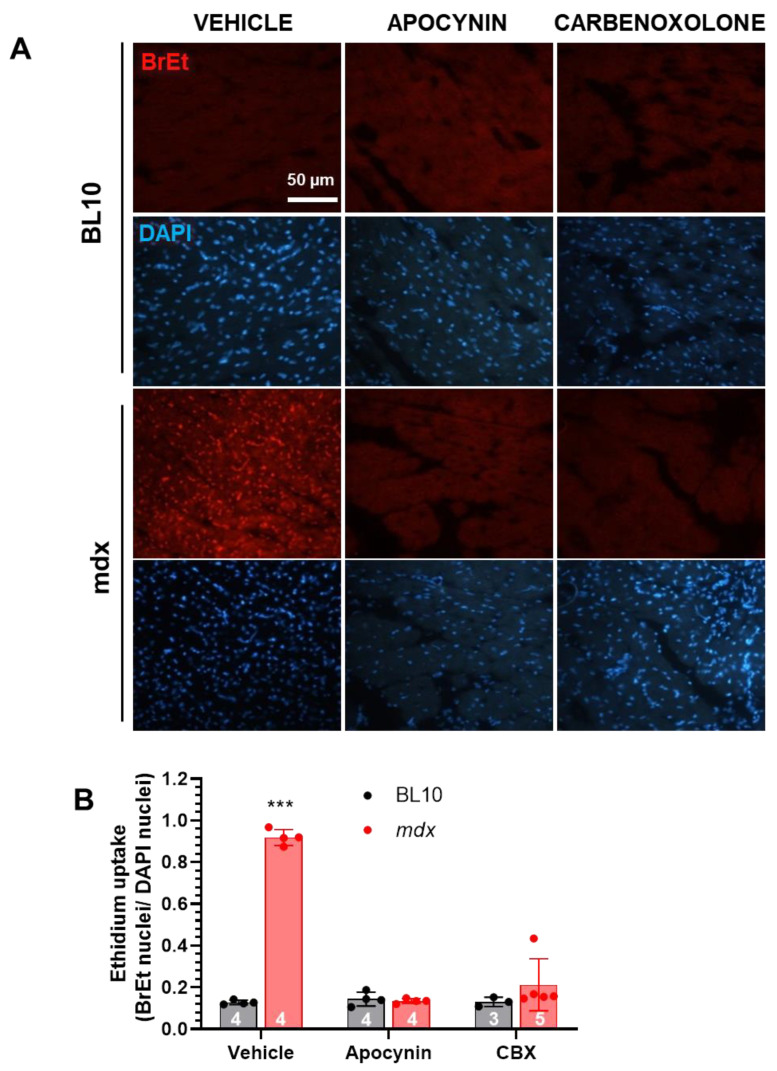
Increased permeability of Cx43 hemichannels in dystrophic hearts. Cardiomyocyte´s permeability was evaluated in Langendorff-perfused hearts using uptake of ethidium (BrEt) as a tracer. (**A**) Representative images of cardiac sections showing ethidium fluorescence (red, top set of panels) and nuclei stained by DAPI (blue, bottom set of panels). The first two columns show control (BL10) and mdx mice treated with the vehicle or apocynin for one month before the perfusion experiment; the third column shows hearts of both strains treated acutely with carbenoxolone (CBX, 10 µmol/L) during perfusion, prior to the ethidium challenge. (**B**) Ethidium uptake quantification, expressed as the ratio of ethidium-positive nuclei to total nuclei. The *n* is indicated in each bar. *** *p* < 0.001 vs. all the other groups, ANOVA with Newman–Keuls as post-hoc test.

Given the presence of lateralized Cx43 in mdx hearts, we hypothesized that at least a fraction of these may correspond to Cx43 hemichannels. To test this point, we designed a protocol to evaluate the permeability of hemichannels by measuring ethidium uptake in isolated Langendorff-perfused hearts, since ethidium can only enter the cells through large pores such as those formed by connexins hemichannels. After 15-min perfusion with ethidium, mdx hearts showed significant nuclear signal for this dye, indicating an increased permeability, as compared with the faint signal in control hearts (Figure 3). Mdx hearts from mice that were treated with apocynin showed minimal ethidium uptake, suggesting that hemichannels activity was dependent on NADPH oxidase-derived oxidative stress. To corroborate that this uptake was due to the presence of Cx43 active hemichannels, control and mdx hearts were treated acutely with carbenoxolone (10 µmol/L), a pharmacological blocker of hemichannels. Treatment with this agent reduced dramatically the permeability to ethidium in mdx hearts, strongly suggesting the presence of active Cx43 hemichannels in the dystrophic hearts.

### 2.4. Apoptosis is Associated to Cx43 Hemichannels Activity

**Figure 4:**
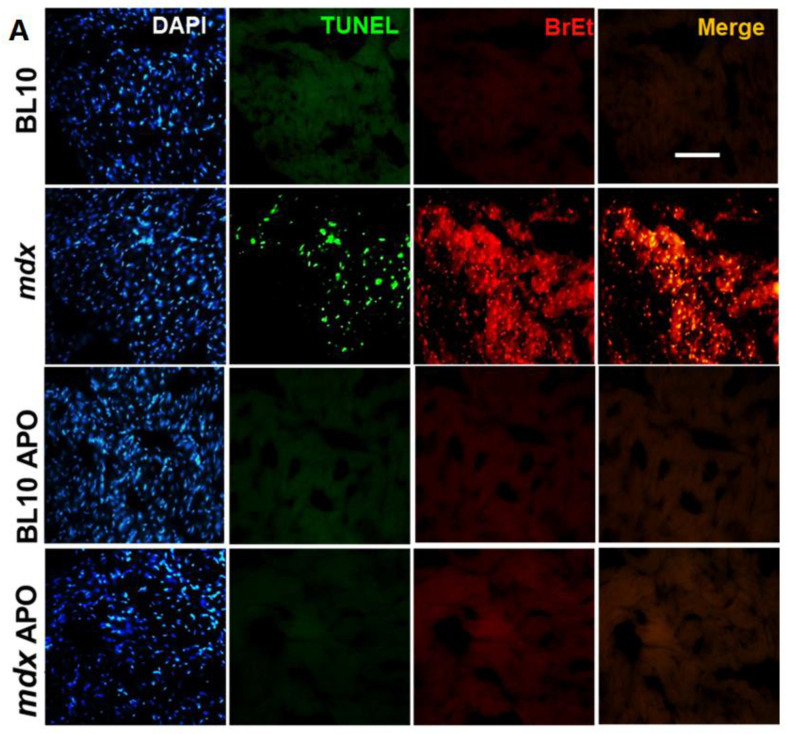

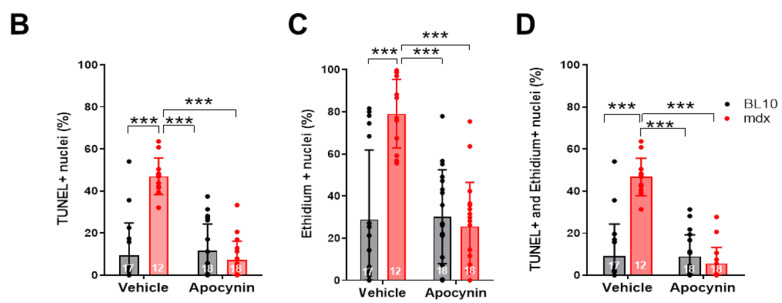
NADPH oxidase inhibition with apocynin reduces apoptosis and permeability in dystrophic hearts. (**A**) Representative fluorescence microscopy images of cardiac sections processed to show total nuclei with DAPI staining (blue), apoptosis with the TUNEL assay (green), hemichannels permeability by ethidium uptake (red) and the merge of TUNEL and ethidium fluorescence. 10-month-old wild type (BL10) and mdx mice that were treated with the vehicle or apocynin (APO) for one month before the perfusion experiment in the isolated hearts were used. The bar indicates 50 µm. (**B**) Quantitative analysis of the images, expressed as percentage of positive TUNEL cells (TUNEL+). (**C**) Percentage of ethidium-positive (Et+) cells and (**D**) percentage of simultaneously TUNEL+ and Et+ cells. *** *p* < 0.001 for the comparisons indicated by the brackets, ANOVA followed by Newman–Keuls post-hoc test. The *n* is indicated in each bar.

In addition to promoting arrhythmias, Cx43 hemichannels may induce cell death by dissipation of ionic gradients and other mechanisms [23]. To evaluate the possibility that cardiomyocytes in mdx hearts are undergoing the process of apoptosis and whether it is associated to hemichannels permeability, we assessed the degree of apoptosis, using the TUNEL assay, in hearts from control and mdx mice that received apocynin or the vehicle. To correlate the increased Cx43 hemichannels activity with apoptosis, we performed the TUNEL assay in hearts that underwent the ethidium uptake protocol and were stained with DAPI (Figure 4A). As hypothesized, this triple staining assay showed that the number of TUNEL and ethidium-positive cells was higher in mdx hearts compared to wild type mice. Furthermore, the treatment with apocynin reduced the number of apoptotic cells significantly. Both the number of TUNEL/ethidium-positive cells and the correlation of TUNEL and ethidium intensity were reduced in the hearts of mdx mice that received apocynin (Figure 4B–D). The quantitative analysis of the images indicates that in the hearts of mdx mice, 47% of the total nuclei were TUNEL+, 79% were Et+ and 47% were both TUNEL and Et+ (Figure 4D). These numbers were reduced to wild type levels when mdx mice were treated with apocynin. These data suggest that cardiomyocytes exhibiting high Cx43 hemichannels activity are undergoing apoptosis or at least are prone to undergo the apoptotic process.

### 2.5. Mechanisms Involved in Cx43 Hemichannels Permeability

#### 2.5.1. Lateralization of Cx43

**Figure 5:**
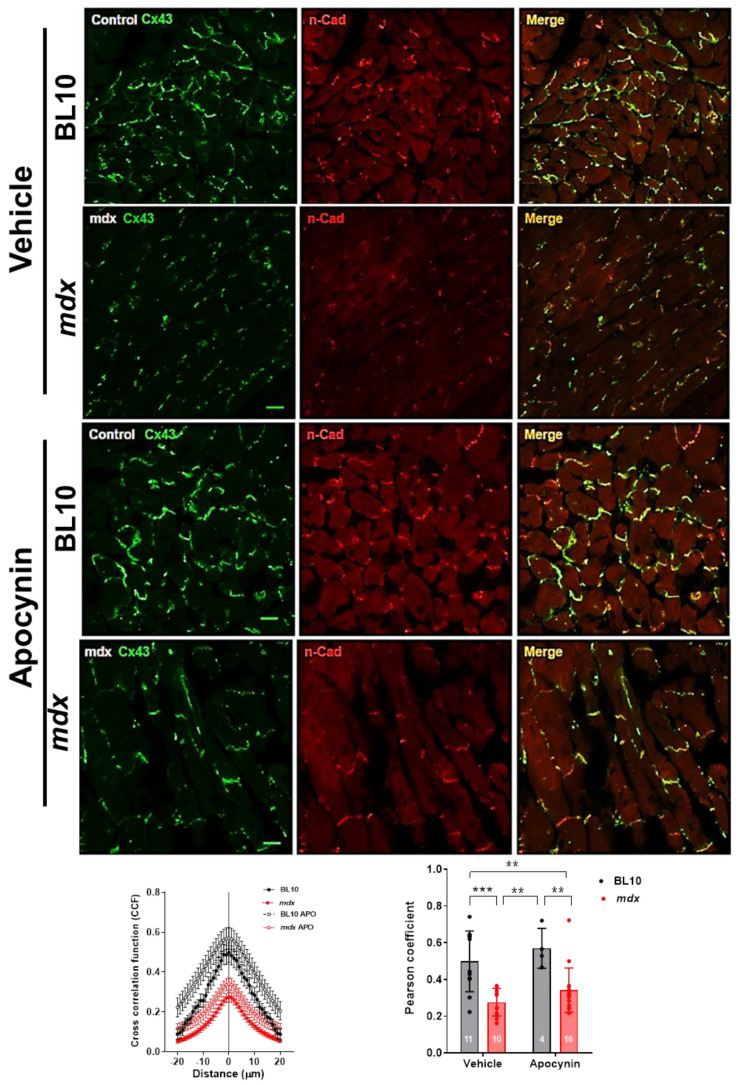
Reduced co-localization of Cx43 and N-cadherin in dystrophic hearts. 10-month-old mdx and their background controls (BL10) mice received the vehicle or apocynin for one month. At the end of this period, hearts were harvested and processed for confocal microscopy. The upper panels show representative immunofluorescence confocal images of cardiac sections for connexin 43 (Cx43, green), N-cadherin (N-Cad, red) and the merge of both signals (co-localization). The bar indicates 10 µm. Below, graphs depicting the analysis of Pearson correlation coefficients obtained for the co-localization of Cx43 and N-cadherin in the confocal microscopy images. ** *p* < 0.005, *** *p* < 0.001 for comparisons indicated by the brackets, ANOVA followed by Newman–Keuls post-hoc test. The *n* is indicated in each bar of the graphs.

We aimed to look into mechanisms that may explain the recovery of cardiac function in mdx mice after the NADPH oxidase inhibition regimen. Oxidative stress, among other effects, has been shown to cause lateralization of connexin 43 in the failing heart [15,24], as we also showed in Figure 1. We decided to investigate whether NADPH oxidase inhibition was able to restore Cx43 location in mdx mice hearts, since lateralized Cx43 forming hemichannels might account for increased permeability and cardiac dysfunction. For this, we performed confocal microscopy and analyzed the degree of co-localization between Cx43 and N-cadherin, a protein of the intercalated discs. In 10-month-old mdx mice, Cx43 was importantly distributed out of the intercalated discs, mainly to the sarcolemma (Figure 5), compared to BL10 control hearts, which show strong co-localization of Cx43 and N-cadherin at the intercalated discs. One-month treatment with apocynin did not change the pattern of Cx43 distribution in mdx hearts, which still showed an important fraction of Cx43 located out of the intercalated discs, reflected in lower values for correlation coefficients between both signals.

**Figure 6:**
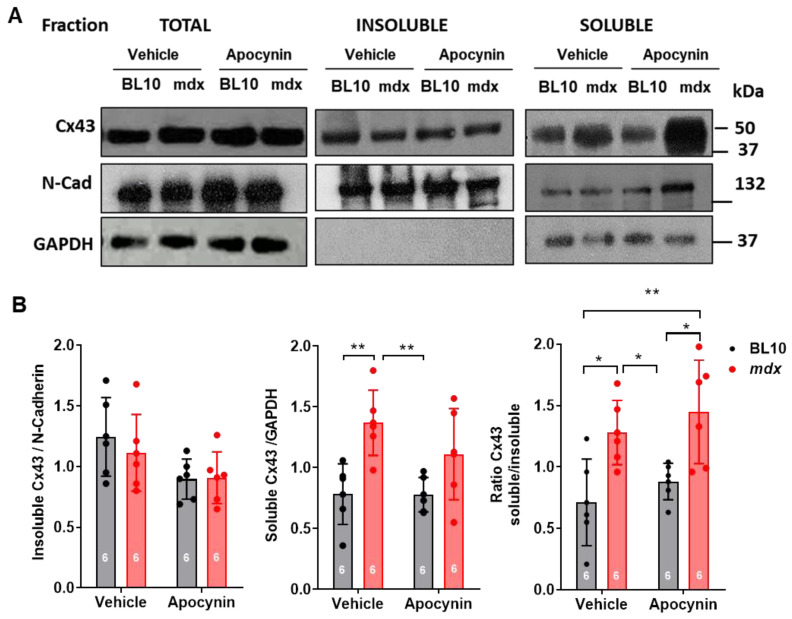
Increased levels of nonjuctional connexin 43 (Cx43) in dystrophic hearts. Controls (BL10) and mdx mice were treated with the vehicle or apocynin for one month. At the end of this period, hearts were harvested for tissue fractionation to separate into soluble (non-junctional) and insoluble (junctional) fractions. Resulting samples of each fraction were probed for connexin 43 (Cx43), N-cadherin (N-Cad) and GAPDH. (**A**) Representative Western blots for Cx43, N-Cad and GAPDH in each fraction. M.W., molecular weight of markers, kDa, kilodaltons. (**B**) Left, graph depicting the ratio of Cx43 insoluble to N-Cad. Middle, ratio of Cx43 to GAPDH in the soluble fraction. Right, ratio of Cx43 soluble to insoluble content. * *p* < 0.05, ** *p* < 0.005 for comparison indicated by brackets, ANOVA with Newman–Keuls as post-hoc test. *n* = 6 hearts in each group.

To further assess this point biochemically, we performed a detergent extraction of cardiac homogenates to separate junctional proteins (intercalated discs) from non-junctional proteins, seeking for lateralized Cx43 in this latter fraction (Figure 6). After following this protocol, it is observed that Cx43 is distributed in both fractions, but the total amount is larger in the insoluble fraction, according to the absolute protein content and load, consistent with an enrichment of this protein in the intercalated discs (Figure 6B). Nevertheless, the intensity of the signal for Cx43 in the soluble fraction was considerably higher in mdx hearts (*p* < 0.005), suggesting that an important amount of Cx43 is located out of the intercalated disc. Again, the treatment with apocynin did not alter this pattern of distribution, or the total amount of Cx43 in mdx hearts, confirming the results obtained by confocal microscopy.

#### 2.5.2. Hyper S-Nitrosylation of Cx43 is Prevented by NOX Inhibition

**Figure 7:**
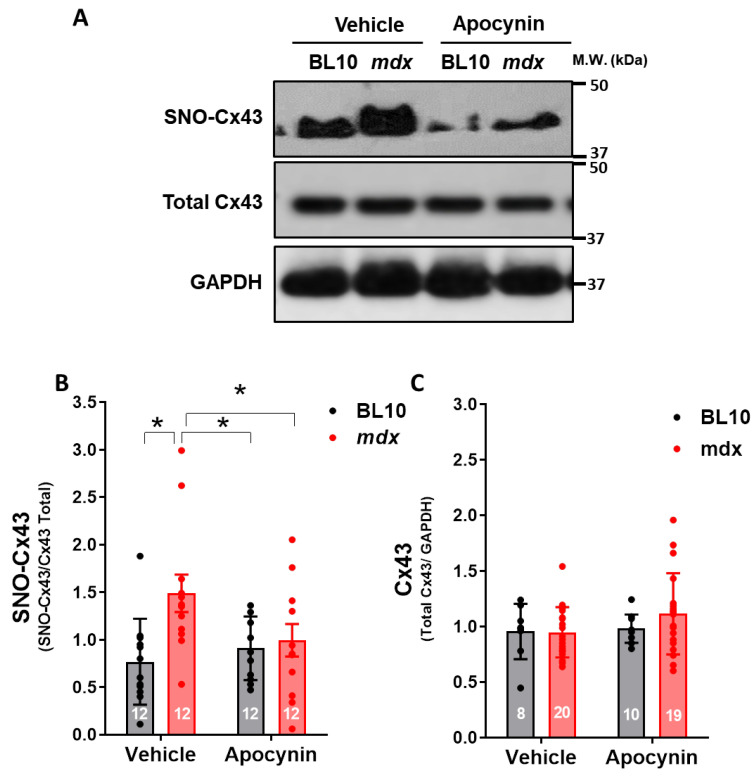
S-nitrosylation of Cx43 is inhibited by NADPH oxidase blockade. (**A**) Representative Western blots for the total and S-nitrosylated (assessed by the biotin-switch technique) levels of Cx43 (SNO-Cx43) in cardiac homogenates from 10-month-old wild type (BL10) and mdx mice, treated with the vehicle or apocynin. All hearts were previously submitted to stimulation with isoproterenol. M.W., molecular weight standards, KDa, kilodaltons. (**B**) Graphs depicting the quantification of Cx43 S-nitrosylation. (**C**) Quantification of the total Cx43 normalized by the levels of GAPDH. *, *p* < 0.05 for the comparisons indicated by brackets, ANOVA followed by Newman–Keuls as post-hoc test. The *n* is indicated inside each bar of the graphs.

Since the levels of lateralized/non-junctional of Cx43 were not altered by the apocynin treatment, in circumstances that this treatment reduced hemichannels activity, we pursued an alternative mechanism that may explain the positive effect of NADPH oxidase inhibition in this model of dystrophic cardiomyopathy. It has been described that oxidative stress induces S-nitrosylation of Cx43, leading to opening of hemichannels [25]. Considering that we found hypernitrosylation of Cx43 in 10-month-old mdx mice hearts, we evaluated this redox modification in the hearts of mice that received apocynin. Using the biotin-switch technique, we confirmed an increased degree of S-nitrosylation in Cx43 in hearts from mdx compared to control mice (Figure 7). Furthermore, Cx43 S-nitrosylation was significantly reduced after treatment with apocynin in mdx mice (*p* < 0.05), suggesting that NOX2-derived oxidative stress and subsequent hemichannel S-nitrosylation are involved in the opening of Cx43 hemichannels in dystrophic hearts. Again, apocynin treatment did not alter the levels of total Cx43 in both strains.

### 2.6. Ventricular Remodeling is Prevented by NADPH Oxidase Inhibition

Finally, we aimed to evaluate the impact of NOX inhibition on ventricular remodeling in 10-month-old mice. Ventricular remodeling is a compensatory mechanism for the increased loss of cardiac function, involving cardiac hypertrophy, development of fibrosis and reduction in cardiac contractility.

**Figure 8:**
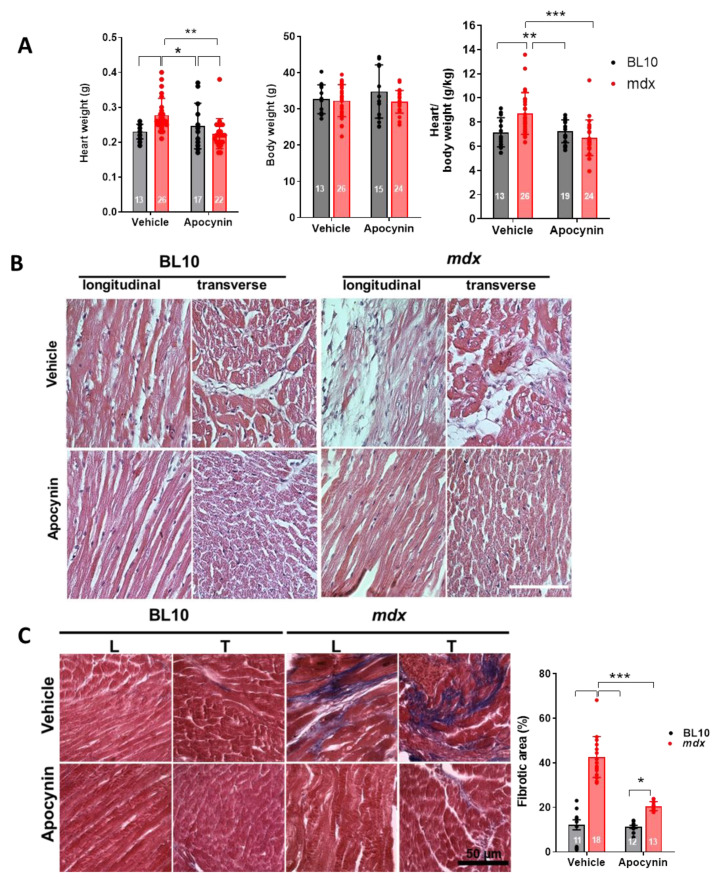
NADPH oxidase inhibition reverses cardiac remodeling in dystrophic hearts. (**A**) Graphs depicting body weight, heart weight and the ratio of heart to body weight in control (BL10) and mdx mice, treated with the vehicle or apocynin for one month. (**B**) Images of hematoxylin and eosin staining for the hearts of 10-month-old control (BL10) and mdx mice treated or not with apocynin, each image representative of 5 hearts. The bar indicates 50 µm. (**C**) Analysis of fibrosis by Masson’s trichrome staining. L, longitudinal sections, T, transverse sections. *, *p* < 0.05, ** *p* < 0.01, *** *p* < 0.001 for the comparisons indicated by the brackets using ANOVA with Newman–Keuls as post-hoc test. The *n* for each group is indicated inside each bar of the graphs.

We found cardiac hypertrophy, evaluated as heart weight and the ratio of heart weight to body weight, (Figure 8A) in mdx mice hearts (8.7 ± 0.3 g/kg) compared to controls (7.2 ± 0.3 g/kg) (*p* < 0.005). However, this hypertrophy was prevented in apocynin-treated mice (7.0 ± 0.2 g/kg in BL10 and 6.7 ± 0.3 g/kg in mdx mice, *p* < 0.05).

Next, we evaluated the impact of apocynin treatment at the histological level in 10-month-old mdx and control hearts. First, hematoxylin and eosin staining revealed a high degree of fiber degeneration and loss of the tissue architecture in mdx hearts. These abnormalities were mostly normalized in mdx hearts from mice treated with apocynin (Figure 8B). Since necrotic cardiomyocytes are replaced by fibrotic tissue, we evaluated the degree of fibrosis by Masson’s trichrome staining (Figure 8B). The hearts from mdx mice presented a higher degree of fibrosis (42.6 ± 2.1% of total area), compared to controls (12.2 ± 2.2%, *p* < 0.001). This fibrotic area was reduced in the apocynin-treated mdx mice (20.5 ± 0.5%, *p* < 0.05). These data suggest that increased activity of Cx43 hemichannels leads to apoptosis, and cardiomyocyte death, ultimately being replaced by fibrotic tissue. Apocynin treatment prevented apoptosis, and thus the development of fibrosis in mdx hearts.

**Figure 9:**
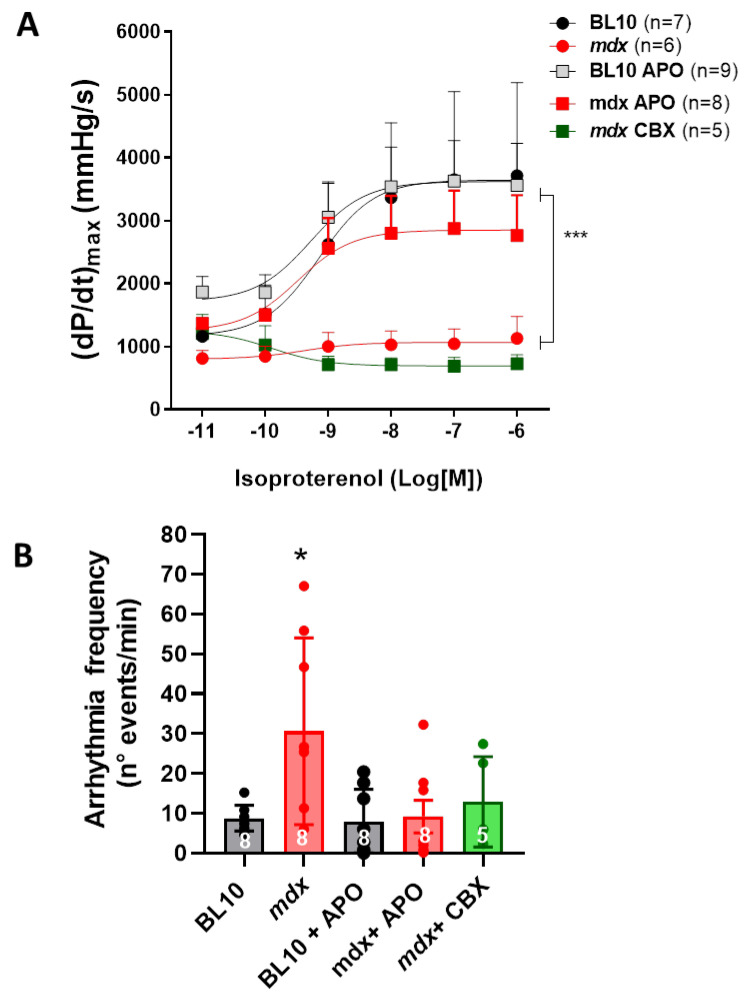
Impact of NADPH oxidase inhibition on cardiac contractility and arrhythmias in dystrophic hearts. (**A**) Graph depicting cardiac contractility evaluated as dP/dt_max_ in response to increasing concentrations of isoproterenol in isolated hearts from 10-month-old control (BL10) and mdx mice, previously treated with the vehicle or apocynin (APO) for one month, or acutely with carbenoxolone (CBX). *** *p* < 0.001 between the curves indicated by the brackets. (**B**) Analysis of the frequency of arrhythmic episodes in the isolated hearts depicted above. *, *p* < 0.005 mdx vs. all the other groups, ANOVA with multiple comparisons followed by Neuman–Keuls as post-hoc test.

Next, we analyzed cardiac parameters in isolated hearts of these mice to investigate whether one month of NADPH oxidase inhibition could reverse or mitigate the changes in cardiac function observed in mdx hearts at this age. Contrary to mdx animals that received the vehicle, mdx mice that received apocynin showed a robust response to isoproterenol, similar to control mice (Figure 9). To explore whether acute Cx43 hemichannel blockade was able to reproduce the restoring effect of NADPH oxidase inhibition, we treated a group of mdx hearts with carbenoxolone. Acute carbenoxolone did not correct the lack of inotropic response to β-adrenergic stimulation.

As mentioned, during the protocol of adrenergic stimulation of isolated hearts, the development of ventricular arrhythmic episodes in 10-month-old mdx hearts in basal conditions became evident, even though, according to the protocol, all hearts were constantly electrically-paced. The frequency of these episodes was dramatically reduced in hearts from mdx mice that were treated with apocynin (Figure 9B), indicating a role for NOX2-derived oxidative stress in mdx electrophysiological disturbances. In addition, acute hemichannel blockade with carbenoxolone suppressed the occurrence of arrhythmic episodes in mdx hearts.

## 3. Discussion

There is accumulating evidence for a central role of NOX-derived oxidative stress in diverse forms of cardiac pathology [9]. The effects of NOX-derived ROS have been shown to impact cardiac biology at several levels such as excitation-contraction coupling, cardiac remodeling, mitochondrial function and metabolism [26]. Here, we document a critical role of NOX-derived ROS in cardiac biology, targeting Cx43 in a mouse model of dystrophic cardiomyopathy. Particularly important in this setting is the activity of Cx43 forming hemichannels. This increased activity of Cx43 hemichannels has a negative impact on cardiac contractility and rhythmicity. In fact, it increased cardiomyocytes apoptosis and produced subsequent fibrosis. In this way, Cx43 hemichannels play an important role in the transition towards cardiomyopathy in mdx mice. NOX inhibition with apocynin abolished the oxidative stress, which in this model became systemic. Our data showed that this maneuver was able to reduce the Cx43-mediated permeability in mdx hearts, suggesting that NOX2-derived oxidative stress activated these pores. Whether this oxidative stress also contribute to Cx43 lateralization is less clear, since NOX inhibition did not restore Cx43 location.

Cx43 hemichannels have been suggested to be activated in acute settings of cardiac damage such as ischemia or metabolic inhibition [19,23,27], but their actual role in vivo or in chronic pathological conditions has remained uncertain. Here, we show that in mdx mice, a model of Duchenne muscular dystrophy, there is a dysregulation of Cx43 that accounts for an important part of the cardiac phenotype of the dystrophic mice. Cx43 is translocated out of the intercalated disc, probably forming hemichannels at lateral membranes. Importantly, the activity of these hemichannels is increased, probably due to hypernitrosylation of Cx43, leading to cardiac dysfunction, and finally apoptosis of cardiac cells. Critical for this process is the increased activity of the superoxide generating system NADPH oxidase. Chronic inhibition of NOX2 reversed this cardiac phenotype, as well as some of these features can be rescued by acute pharmacological inhibition of Cx43 hemichannels.

The mechanism by which NOX2 activity results in increased Cx43 S-nitrosylation is not clear. It has been suggested that in mdx mice, transient receptor potential canonical channel 6 (Trpc6) induces an increase in the S-nitrosylation proteome [28], but the exact mechanisms remain unknown. TRPC channels may promote Ca^2+^ entrance that activates nitric oxide synthase and NO production. However, in turn, TRPC channels, particularly TRPC3, are also regulated by NOX2 activity, raising the possibility that NADPH oxidase inhibition ultimately results in reduced S-nitrosylation [28]. Interestingly, these authors reported in their proteomic analysis a dramatic increase in the degree of S-nitrosylation of Cx43 in mdx-utrophin deficient mice, a more acute model of Duchenne dystrophy [28]. More precisely, they reported a nine-fold increase in the S-nitrosylation of Cys 271 of Cx43 compared to control mice, which is consistent with a previous report of Cx43 nitrosylation [29].

Although we did not determine whether S-nitrosylated Cx43 corresponded to junctional or hemichannels, it has been previously reported in astrocytes under metabolic inhibition the increased nitrosylation of Cx43 that forms hemichannels [25]. Very recently, while this manuscript was in preparation, Lillo et al. elegantly showed that S-nitrosylation at Cys 271 directly activates Cx43 hemichannels, and that NOS inhibition in mdx mice prevented the occurrence of arrhythmic episodes along with preventing Cx43 hemichannel S-nitrosylation [30]. Interestingly, these authors reported that arrhythmic events are present only in β-adrenergic stimulated mdx hearts as S-nitrosylation is increased by isoproterenol (1 µmol/L). Our results indicate that isolated mdx hearts have a significant incidence of arrhythmic episodes, both under basal conditions and during adrenergic stimulation with isoproterenol. Additionally, our results demonstrate that increased ROS production by NADPH oxidase is required for Cx43 S-nitrosylation, and complete prevention of arrhythmic episodes is achieved in apocynin-treated mdx mice, even though NOS isoforms were not inhibited. This is in line with the observation that Ca^2+^-handling proteins are S-nitrosylated in response to isoproterenol, in a process dependent on nNOS function, but without evidence of an increase in its activity [31].

Physiologically, NOX2 has been shown to be mechanosensitive and is upregulated in dystrophic hearts [13]. The mechanism for this upregulation includes regulation by microRNAs [32] and NOX2 stabilization by TRPC3 [33].

The observations presented here open a new line of research for the role of Cx43 hemichannels in cardiac pathologies, beyond ischemic related injuries, in which Cx43 has been extensively reported to play a role [19,27,34,35]. Our findings are consistent with those of Gonzalez et al., who showed that mdx animals presented arrhythmias induced by isoproterenol that were corrected by Cx43 blocking peptides [36]. Furthermore, our results go beyond the acute effect of Cx43 hemichannels and show that these hemichannels are involved in the whole process of cardiac remodeling and dysfunction in dystrophic hearts. This role for Cx43 hemichannels in the development of cardiac failure has long been suspected [37], as lateral Cx43 distribution has been observed in several types of cardiac disease, including dystrophic cardiomyopathy [36,38].

Indeed, it is possible that the effects of reducing oxidative stress in mdx hearts by inhibition of NADPH oxidase with apocynin may affect other targets beside Cx43 hemichannels. These include other redox-sensitive proteins such as the ryanodine receptor (RyR2) [39,40]. Our group has described that apocynin restores the impaired contractility of mdx mice and in a model of aging by restoring phospholamban phosphorylation [11,41]. Nevertheless, the importance of Cx43 hemichannels in the progression of the dystrophic cardiomyopathy is evidenced by fact that reducing hemichannels permeability reduced the degree of apoptosis in cardiac cells of the mdx hearts, resulting in decreased fibrosis and cardiac hypertrophy. NOX inhibition reduced oxidative stress in mdx hearts, but was unable to reverse the degree of lateralization of Cx43. Interestingly, despite this, prevention of oxidative stress by inhibition of NOX reduced Cx43 hemichannels activity, probably by decreasing the degree of S-nitrosylation. Prevention of oxidative stress was unable to correct the location of Cx43 back to intercalated discs, probably due to the profound alterations in the structure of the cytoskeleton in dystrophic cardiomyocytes. It has been shown that the microtubule lattice is completely disorganized in mdx myocytes [42,43] and microtubules are the highways for targeting Cx43 to the intercalated discs [44]. A recent report indicates that Cx43 hypo-phosphorylation is the cause for Cx43 connexons to be misplaced to the plasmalemma, forming hemichannels susceptible for opening [45]. Furthermore, these authors propose a redundant circle in which increased hemichannel activity causes increased NADPH oxidase production of ROS.

In this study, we used carbenoxolone as a hemichannel blocker. Carbenoxolone is a preferential Cx hemichannels blocker that can also block pannexin (Px) channels [46]. In this sense, it is important to note that there are several pathological situations where dysregulation of Cx hemichannels seems to concur with increased activity of (Px) channels [46,47,48,49]. The interaction between both types of channels is complex, inasmuch in so many cases, blockade of one or the other type of pore-forming hexamers results in abrogation of the overall cellular hyperpermeability, in a non-additive process [50,51]. These observations cannot be explained by eventual un-specificity of pharmacological blockers, since use of specific peptides produces similar effects as using more general chemical blockers as that used in this study; instead, they probably reveal a complex reiterative loop in which hemichannels activity of one type causes opening of the other kind. A full dose–response study cannot be carried out in the beating heart because at higher concentrations, hemichannels blockers do interfere with Cx forming GJ channels mediated cardiac conduction, causing heart arrest.

### Limitations of the Study

Obviously, this study presents several limitations. These, among others, arise from the lack of specificity of apocynin as NOX inhibitor, which precludes discarding other sources of oxidative stress from contributing to this pathophysiological mechanism. Furthermore, our observations come from ex vivo experiments, not from in vivo data, which requires other kinds of instrumentation.

## 4. Materials and Methods

### 4.1. Animals

Male C57BL/10ScSn-DMDmdx/J (mdx) and control mice (C57BL/10SnJ) (BL10) were purchased from Jackson Laboratories (Bar Harbor, MA, USA), maintained and bred at the animal facility of the Pontificia Universidad Catolica de Chile (Santiago, Chile). All the experimental protocols were performed on 2- and 10-month-old mice and were approved by the Institutional Committee of Bioethics and Bio-Safety, and the Committee of Bioethics, Universidad de Talca N°2015-03-A from 12 November 2015 and Pontificia Universidad Catolica de Chile (CBB-156/2011) and conformed to the Guide for the Care and Use of Laboratory Animals published by the US National Institutes of Health (NIH publication No. 85–23, revised 1996). Groups of BL10 and mdx mice were randomized when nine months old to receive either apocynin (40 mg/kg/day) or the vehicle (ethanol) in drinking water for one month, as previously described [52,53]. These mice were studied at 10 months of age.

### 4.2. Isolated Heart Preparation

Mice were anesthetized with a mixture of ketamine (90 mg/kg) and xylazine (10 mg/kg) and pre-medicated with 1000 IU of heparin i.p. Hearts were rapidly excised and perfused through the aorta with Krebs–Henseleit buffer (equilibrated with a gas mixture of 95% O_2_ and 5% CO_2_ at 37°C) using a peristaltic pump. A polyvinyl chloride balloon connected to a pressure transducer was placed through the left atrium and mitral valve into the left ventricle. The balloon was filled with saline to determine isovolumetric intraventricular pressure. Perfusion flow was increased gradually until reaching 4 mL/min. The hearts were placed in a heated chamber and paced at 500 beats/min with platinum electrodes using a Grass stimulator (Grass Instruments, Quincy, MA, USA) (pulses of 5 V, 1 ms). Left ventricular pressure (LVP) and coronary perfusion pressure (CPP) were measured continuously with pressure transducers and digitized to obtain the rate of change in left ventricular pressure (dP/dt). Minimal diastolic pressure was held constant at 5–10 mm Hg during the experiment. After an equilibration period (10 min), hearts were perfused with increasing concentrations of isoproterenol (0.1 nmol/L −1.0 µmol/L, 3 min each).

### 4.3. Western Blotting

Hearts were rapidly excised and perfused through the aorta with Krebs–Henseleit buffer. After an equilibration period (10 min), hearts were quickly removed and ventricular tissue was homogenized in 0.5 mL of cold HEN buffer (HEPES 250 mM, EDTA 1mM y neocuproine 0.1 mmol/L, pH 7.4) containing proteases and phosphatases inhibitors (leupeptine 10 mg/mL, pepstatin A 10 mg/mL, benzamidine 1 mmol/L, PMSF 1 mmol/L, sodium fluoride 50 mmol/L and sodium orthovanadate 1 mmol/L) using an Ultra-Turrax (T8 IKA Labortechnil IKA-Werke GmbH & Co, KG, Staufen, Germany). Homogenates were centrifuged at 5000 rpm for 10 min. The supernatant was recovered and 40 μL/mL 10% CHAPS was added. Protein concentration was determined and the samples were mixed with Laemli 4× buffer containing β-mercaptoethanol and boiled. Proteins were resolved in a 10% SDS-PAGE, electroblotted to a nitrocellulose membrane and stained with Ponceau red to evaluate the total protein content. Membranes were blocked with Tween 20-PBS solution supplemented with 5% skim milk. Then, proteins were incubated with mouse monoclonal anti-Cx43 (1:1000), source anti-GADPH (1:000), rabbit polyclonal anti-N-cadherin (1:750) and source anti-p22 (1:500) (Santa Cruz Biotechnology, Santa Cruz, CA, USA) overnight. After washing for 1 h with Tween 20-PBS buffer, membranes were incubated with either anti-mouse or anti-rabbit IgG peroxidase conjugated antibodies (1:5000) and washed for 1 h. Finally, proteins were visualized by chemiluminescence. For all Western blot analysis, the intensity of the signal was evaluated using the ImageJ program (NIH).

### 4.4. Quantification of S-Nitrosylated Proteins

Hearts were rapidly excised, perfused and equilibrated and homogenized as above. S-nitrosylated proteins were detected by the biotin-switch method as previously described [31]. First, free thiols (-SH) in tissue homogenates were blocked for 1 h at 50°C in the dark with methyl methanethiosulfonate (MMTS). Proteins were precipitated with 4 volumes of ice-cold acetone, washed repeatedly with acetone to remove free MMTS and resolubilized. Thereafter, nitrosylated cysteine residues (-SNO) were reduced to free cysteine by incubating 1 h with 30 mM sodium ascorbate and selectively labeled with HPDP-biotin. Proteins were precipitated again with acetone to wash the excess of HPDP-biotin and solubilized with HENS buffer (HEN buffer with 1% SDS). Following solubilization, the samples were incubated 1 h with agarose-conjugated streptavidin beads (Sigma-Aldrich Chemical, St Louis, MO, USA) and centrifuged to pull HPDP-biotinylated proteins down. Adsorbed proteins were resolved in a 10% SDS-PAGE, electroblotted to a nitrocellulose membrane and stained with Ponceau red to evaluate the total protein content. Then, the biotinylated proteins were probed with anti-Cx43 (Sigma Aldrich Chemical, St Louis, MO, USA) antibodies. For all Western blot analysis, the intensity of the signal was evaluated using the ImageJ program (NIH public domain software).

### 4.5. Extraction of Non-Junctional and Junctional Proteins

To separate gap junctions Cx43 (the “junctional” fraction) from elsewhere in the cell (the “non-junctional” fraction), we performed a Triton X-100 extraction protocol [54]. Ventricular tissue was homogenized in 0.5 mL of cold extraction buffer (25 mmol/L Tris, 150 mmol/L NaCl, 1% Triton, pH 7.4) with proteases and phosphatases inhibitors. The samples were incubated on ice for 30 min, with vortexing every 5 min, and then centrifuged (15,000 × 30 min, 4°C). Non- junctional proteins from the supernatant were collected. Junctional proteins in the pellet were re-suspended in an equal volume of extraction buffer containing 4 mol/L urea. Samples were incubated at room temperature for 30 min with constant vortexing, centrifuged (15,000 rpm, 30 min, 4°C) and junctional proteins were collected from the supernatant.

### 4.6. Tissue Preparation for Histological Analysis

Hearts were rapidly excised and perfused through the aorta with Krebs–Henseleit buffer to remove remaining blood. Ventricular tissue was fixed overnight in Bouin solution, embedded in paraffin and processed for hematoxylin–eosin, TUNEL assays (Roche Diagnostics; Mannheim, Germany) and Masson trichrome staining (Sigma-Aldrich Chemicals, St Louis, MO, USA).

### 4.7. Immunofluorescence

Heart sections (8 µm) were blocked at room temperature (1 h) with 3% PBS/BSA, then the slides were incubated with mouse anti-Cx43 (1:200) and rabbit anti-*N*-cadherin (1:50;) in 3% PBS/BSA at 4 °C overnight. Following several washes with PBS/tween 0.1%, slides were incubated for two hours at room temperature with Alexa 488 conjugated anti-mouse (Invitrogen Thermo Fisher Scientific, Carlsbad, CA, USA) and Alexa 568 conjugated anti-rabbit antibodies. Slides were washed with PBS/Tween 0.1%, counterstained with propidium iodide 3 µmol/L for 10 min to visualize the nuclei, mounted with Fluoromont (Sigma-Aldrich Chemical, St Louis, MO, USA), analyzed with a BX5I confocal microscope (Olympus, Center Valley, PA, USA) and photographed with a PM-30 digital camera (Olympus Corporation, Tokyo, Japan). The degree of colocalization between Cx43 and N-cadherin was calculated using Pearson coefficient and Van Steensel coefficient [55,56] by the ImageJ program (NIH public domain software).

### 4.8. Ethidium Uptake Assay

Hearts were rapidly excised and perfused through the aorta with Krebs–Henseleit buffer. After an equilibration period (10-min), hearts were treated with the vehicle or hemichannel blocker carbenoxolone (10 µmol/L, Sigma-Aldrich Chemical, St Louis, MO, USA) for 10 min. After this period, hearts were perfused with ethidium bromide (BrEt) 15 µmol/L (Sigma-Aldrich Chemical, St Louis, MO, USA) for 15 min, in the same medium (with or without blocker) followed by a 15-min wash period. Then, hearts were embedded in 2-methylbutane and frozen in liquid nitrogen. Cryosections (8 μm) were obtained, washed with PBS and fixed with 4% paraformaldehyde. After fixation, the sections were washed with PBS, mounted using Prolong gold antifade reagent containing 4′,6-diamidino-2-phenylindole (DAPI) (Invitrogen, Thermo Fisher Scientific, Carlsbad, CA, USA), observed with a BX41 Olympus fluorescence microscope and photographed with a ProgRes C5 digital camera (Olympus Corporation, Tokyo, Japan). The intensity of the signal was evaluated using the ImageJ program (NIH public domain software).

### 4.9. NADPH Oxidase Activity

NADPH oxidase activity was measured indirectly as the reduction of cytochrome C by superoxide (O_2_^•ˉ^) in the presence of NADPH. For this assay, heart homogenates were centrifuged at 1000 rpm for 10 min at 4 °C. After this, the supernatants were removed and centrifuged at 12,000 rpm for 10 min at 4 °C. The resulting supernatants were mixed with working solution (300 mmol/L KH_2_PO_4_, 0.1 mmol/L EDTA, 36 μmol/L cytochrome C, pH 7.8 and 20 μL of 50 mmol/L KCN and 100 μL of NADPH solution (40 mg/mL). Seven absorbance measurements at 550 nm were performed, from 0–6 min. Values obtained were finally corrected by the protein concentration of each sample.

### 4.10. TBARS Measurement

Levels of thiobarbituric acid reactive substances (TBARS) were estimated using the method described by Ramanathan et al. [57] with slight modifications. Heart homogenates supernatants were mixed with SDS (8% *w*/*v*), TBA (0.8% *w*/*v*) and acetic acid (20% *v*/*v*) and heated for 60 min at 90 °C. Precipitated material was removed by centrifugation, and the absorbance at 532 nm of the supernatant was determined. Levels of TBARS were calculated using a calibration curve with malondialdehyde as standard (MDA, Sigma-Aldrich Chemical, Saint Louis, MO, USA).

### 4.11. TUNEL Assay

Paraffin-included hearts were cut into sections (8 µm), and blocked at room temperature (1 h) in PBS 3% BSA and 0.1% Tween 20. Then, the sections were incubated for 1 h in the dark with the TUNEL reagent according to the manufacturer´s instructions (Roche Diagnostics GmBlt, Mannheim, Germany). After this period, sections were washed 3 times for 10 min with PBS and then incubated with DAPI (3 μmol/L) for 10 min. After this, sections were washed 3 times with PBS for 10 min and then mounted with Flouromount. For the case of frozen samples, first, they were fixed in paraformaldehyde 4% during 10 min at room temperature. Then, the slides were washed 3 times with PBS/glycine (1.5 mg/mL) for 10 min. After this, the sections were immersed in sodium citrate 0.01 mol/L, and heated to boiling temperature. Then, the sections were blocked with 3% PBS/BSA Tween 0.1% during 1 h at room temperature. After blocking, the sections were processed similarly to paraffin-fixed samples.

### 4.12. Statistical Analysis

Data are presented as means ± standard deviation (S.D.), except concentration response curves where standard error is presented. Data from concentration–response experiments were fitted to a sigmoid equation as previously described [41]. Differences between groups were analyzed by two-way ANOVA, using Newman–Keuls as post-hoc test. Graphpad Prism 8.0.2 software (Graphpad Software Inc., San Diego, CA, USA) was used for these analyses. A value of *p* < 0.05 was considered statistically significant.

## 5. Conclusions

Taken together, our results suggest that, during DMD pathogenesis, increased NADPH oxidase activity induces S-nitrosylation of cardiac Cx43 already redistributed from intercalated discs to the plasma membrane, thus causing formation and/or activation of Cx43 hemichannels that contribute to cardiac dysfunction and apoptosis.

In summary, we have presented evidence that in the heart of mdx mice, Cx43 forms functional hemichannels that contribute to the progression of the disease. Importantly, this occurs in the context of increased oxidative stress derived from NADPH oxidase.

## Abbreviations

Cx43	Connexin 43
NOX	NADPH oxidase
